# Supplementary figures and images for: Microbiome Features Differentiating Unsupervised-Stratification-Based Clusters of Patients with Abnormal Glycometabolism
Source: mBio. 2023 Jan 18;14(1):e03487-22. doi: 10.1128/mbio.03487-22 (PMC9973283; doi:10.1128/mbio.03487-22)

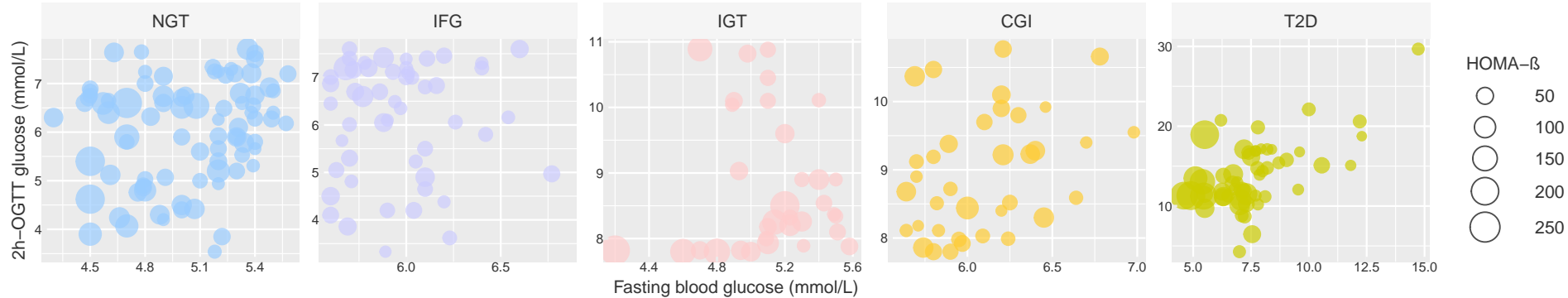

Supplement: FIG S1 [file mbio.03487-22-s0001.pdf]

Shannon index

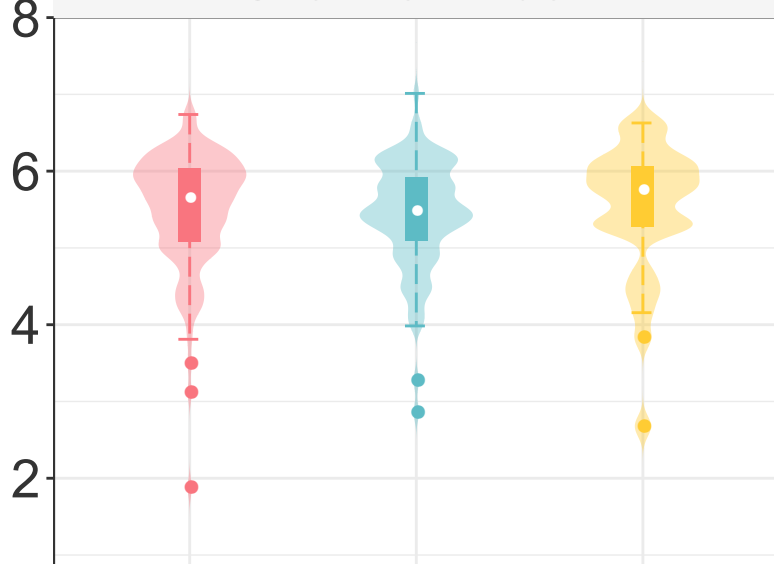

Simpson index

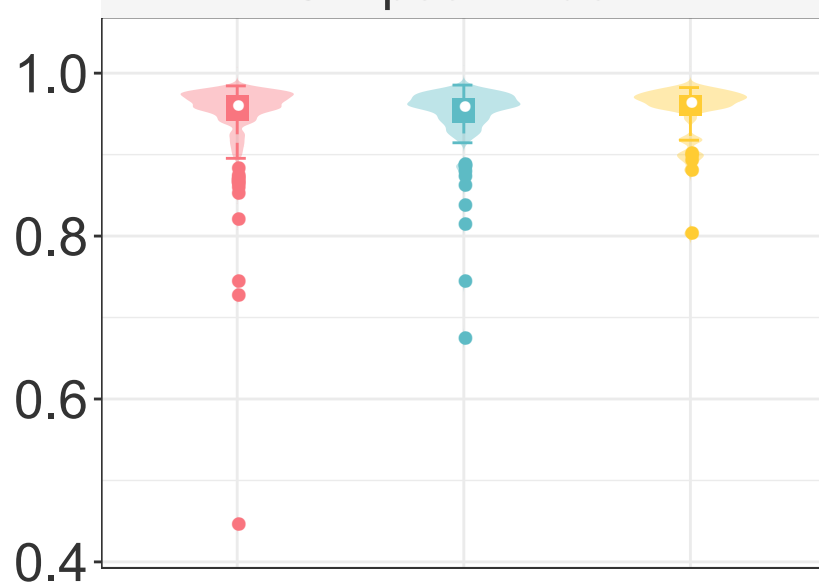

Observed ASVs

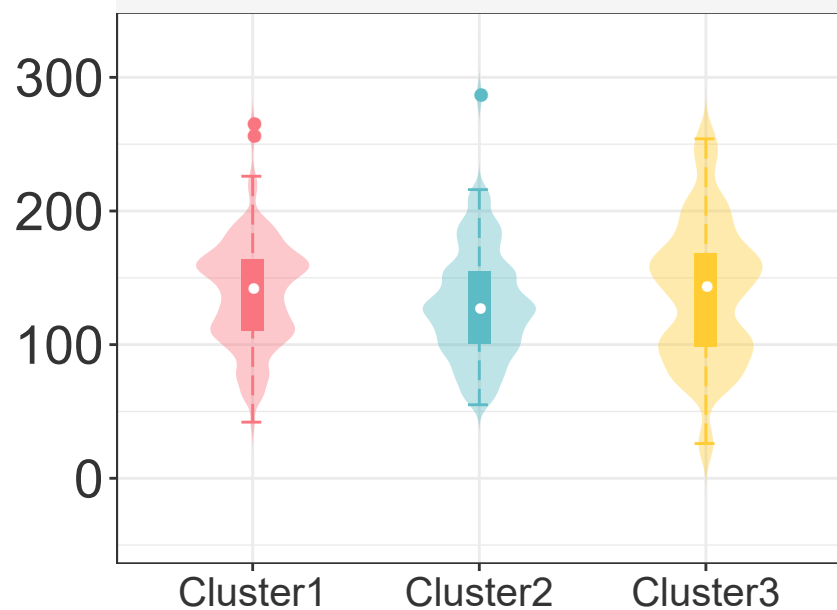

PD whole tree

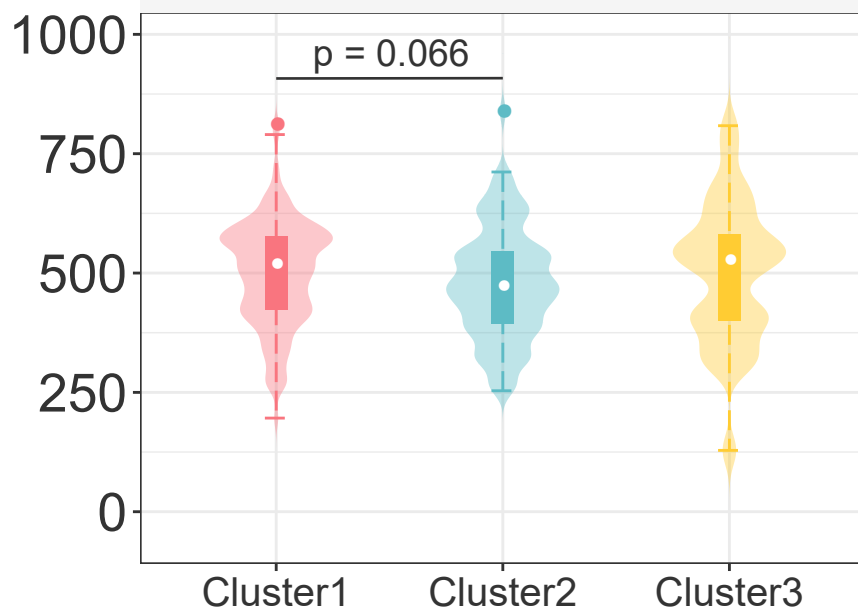

Supplement: FIG S2 [file mbio.03487-22-s0002.pdf]

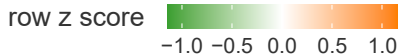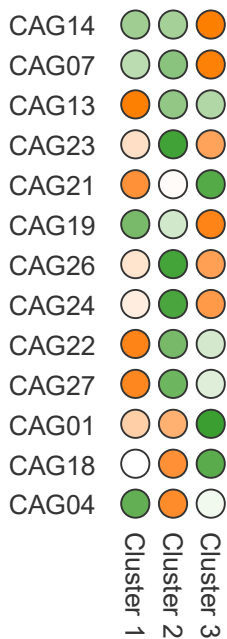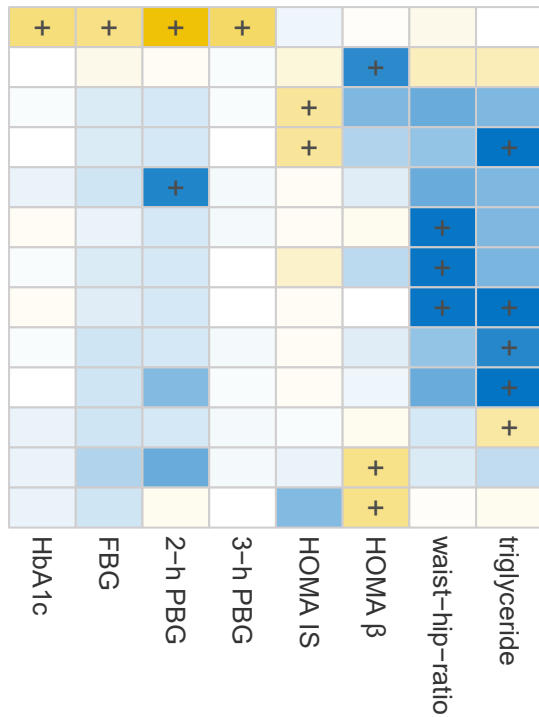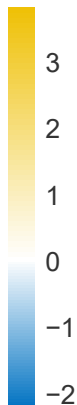

Supplement: FIG S3 [file mbio.03487-22-s0003.pdf]

A

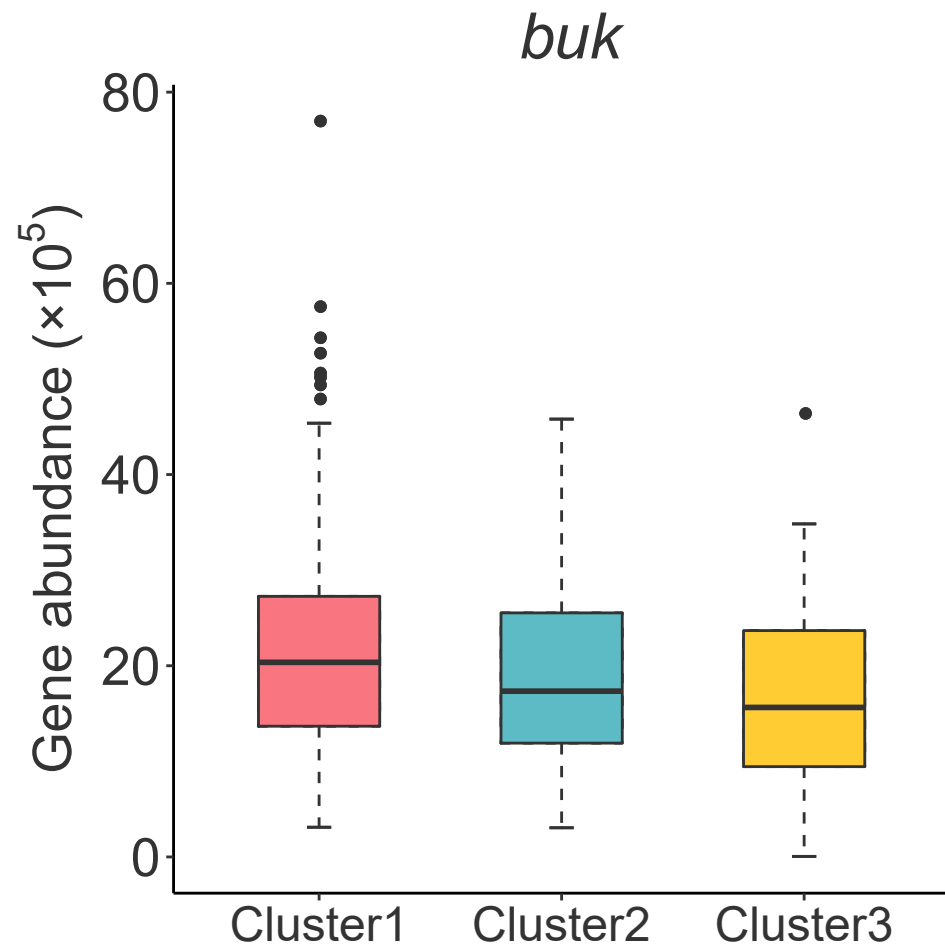

B

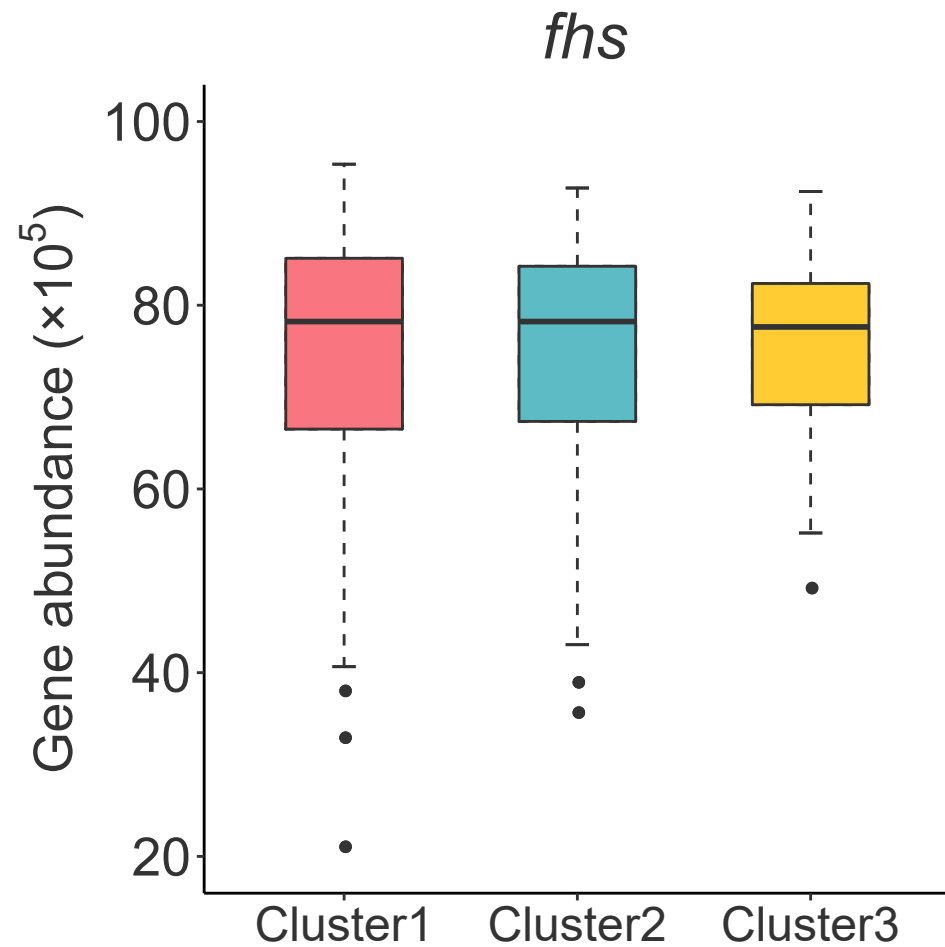

Supplement: FIG S4 [file mbio.03487-22-s0004.pdf]

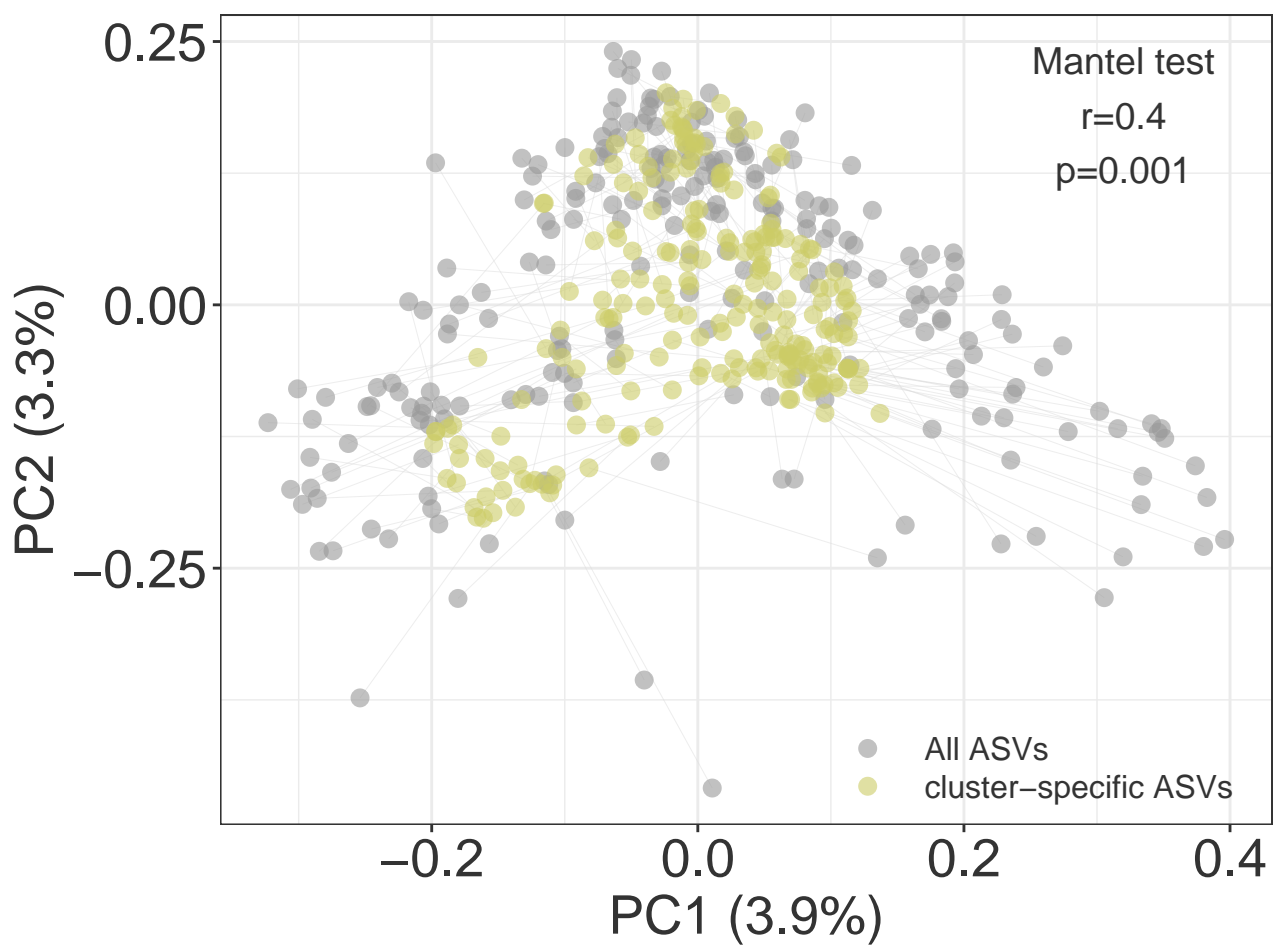

Supplement: FIG S5 [file mbio.03487-22-s0005.pdf]

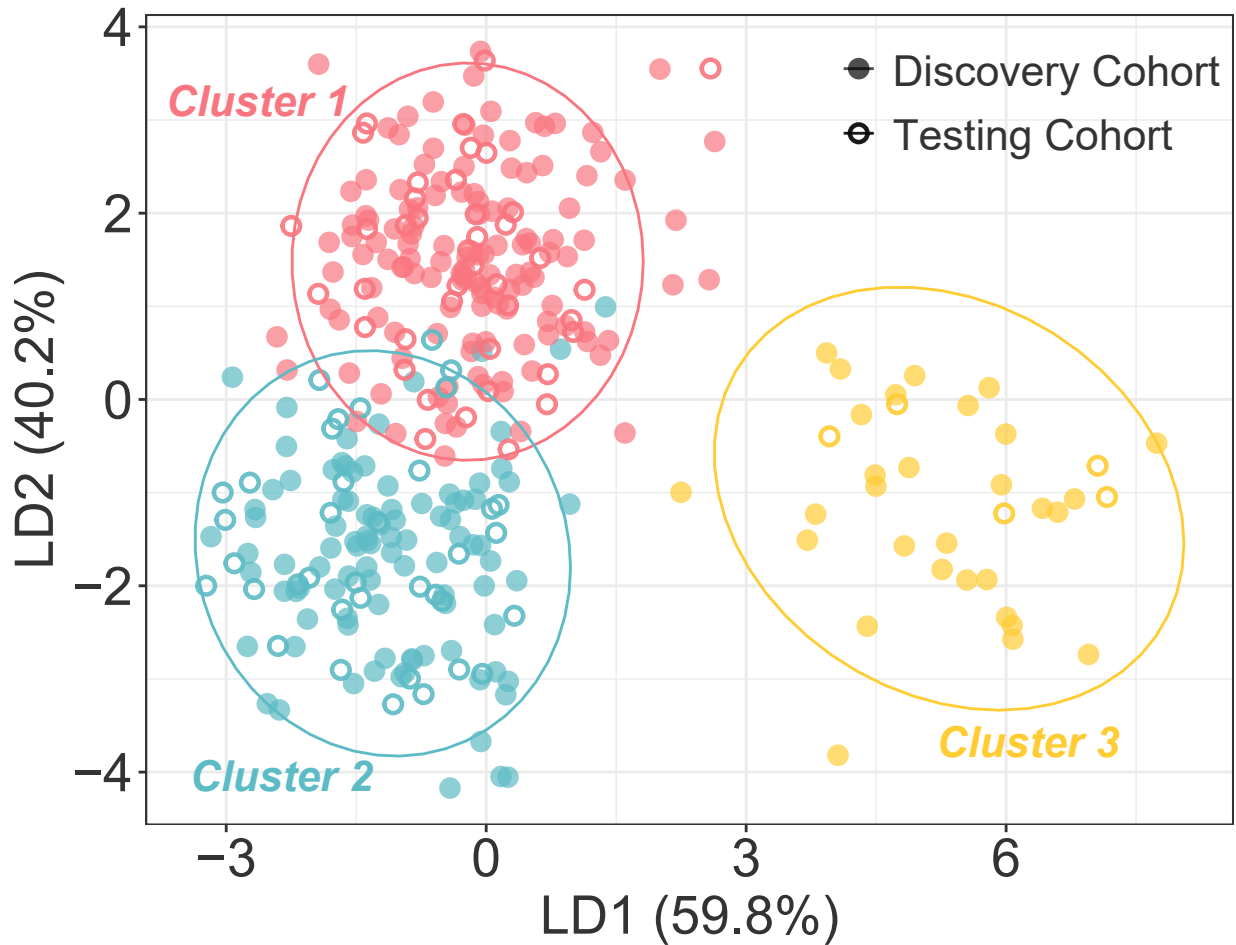

Supplement: FIG S6 [file mbio.03487-22-s0006.pdf]
