# Supplementary material for: Microbiome Features Differentiating Unsupervised-Stratification-Based Clusters of Patients with Abnormal Glycometabolism
Source: mBio. 2023 Jan 18;14(1):e03487-22. doi: 10.1128/mbio.03487-22 (PMC9973283; doi:10.1128/mbio.03487-22)
Supplement: TABLE S2 [file mbio.03487-22-s0008.docx]

**Table S2 Comparisons of clinical parameters among clusters in testing cohort**

|  | Cluster1 | Cluster2 | Cluster3 | *P*-value |
| --- | --- | --- | --- | --- |
| **General Information** |  |  |  |  |
| Age (years) | 53.85 ± 0.71 (41) | 53.49 ± 0.84 (37) | 53 ± 1.87 (5) | 8.7E-01 |
| Gender (F/M) | 25/16 | 19/18 | 2/3 | ns |
| **Glycometabolism indicators** |  |  |  |  |
| HbA1c (%) | 5.45 ± 0.05 (41) | 5.55 ± 0.04 (37) | 5.76 ± 0.26 (5) | 4.9E-02 |
| FBG (mmol/L) | 6.04 ± 0.07**^a^** (41) | 6.13 ± 0.07**^a^** (37) | 7.4 ± 0.39**^b^** (5) | 1.8E-03 |
| 0.5-h PBG (mmol/L) | 9.59 ± 0.29**^a^** (41) | 10.51 ± 0.23**^b^** (37) | 12.19 ± 1**^b^** (5) | 3.6E-03 |
| 1-h PBG (mmol/L) | 9.1 ± 0.35**^a^** (41) | 10.42 ± 0.43**^b^** (37) | 14.33 ± 0.67**^c^** (5) | 2.2E-04 |
| 2-h PBG (mmol/L) | 6.77 ± 0.22 (41) | 7.63 ± 0.29 (37) | 9.47 ± 1.33 (5) | 1.6E-02 |
| glucose AUC (mmol/L/min) | 990.95 ± 24.02**^a^** (41) | 1105.27 ± 28.28**^b^** (37) | 1405.89 ± 98.35**^c^** (5) | 7.5E-05 |
| HOMA-IR | 1.18 ± 0.08**^a^** (41) | 1.85 ± 0.17**^b^** (37) | 1.39 ± 0.33**^ab^** (5) | 4.6E-04 |
| HOMA-IS | 1.05 ± 0.09**^a^** (41) | 0.72 ± 0.09**^b^** (37) | 1 ± 0.34**^ab^** (5) | 4.6E-04 |
| HOMA-β | 35.21 ± 2.43**^a^** (41) | 51.01 ± 3.82**^b^** (37) | 23.24 ± 7.42**^a^** (5) | 3.3E-04 |
| fasting insulin (μU/mL) | 4.37 ± 0.28**^a^** (41) | 6.7 ± 0.56**^b^** (37) | 4.28 ± 1.12**^ab^** (5) | 2.5E-04 |
| 0.5-h insulin (μU/mL) | 43.21 ± 3.29**^a^** (41) | 70.71 ± 5.27**^b^** (37) | 20.07 ± 3.12**^c^** (5) | 1.6E-06 |
| 1-h insulin (μU/mL) | 40.38 ± 3.13**^a^** (41) | 78.02 ± 8.48**^b^** (37) | 37.36 ± 10.53**^a^** (5) | 1.2E-04 |
| 2-h insulin (μU/mL) | 27.66 ± 2.26**^a^** (41) | 55.99 ± 5.46**^b^** (37) | 41.09 ± 16.33**^ab^** (5) | 3.0E-04 |
| insulin AUC (μU/mL/min) | 4009.16 ± 263.74**^a^** (41) | 7412.08 ± 579.24**^b^** (37) | 3580.19 ± 1027.5**^a^** (5) | 1.7E-06 |
| **Anthropometric markers** |  |  |  |  |
| BMI (kg/m2) | 24.54 ± 0.34**^a^** (41) | 27.07 ± 0.39**^b^** (37) | 25.12 ± 1.22**^ab^** (5) | 4.4E-05 |
| height (cm) | 158.29 ± 1.09 (41) | 159.07 ± 1.48 (37) | 158.5 ± 3.94 (5) | 1.0E+00 |
| weight (kg) | 61.6 ± 1.18**^a^** (41) | 68.71 ± 1.65**^b^** (37) | 62.9 ± 2.72**^ab^** (5) | 5.0E-03 |
| waist circumference (cm) | 80.2 ± 0.98**^a^** (41) | 90.3 ± 1.08**^b^** (37) | 87 ± 2.39**^ab^** (5) | 5.4E-07 |
| hip circumference (cm) | 94.73 ± 0.59**^a^** (41) | 100.62 ± 0.92**^b^** (37) | 95.6 ± 1.5**^ab^** (5) | 2.2E-05 |
| waist-to-hip ratio | 0.85 ± 0.01**^a^** (41) | 0.9 ± 0.01**^b^** (37) | 0.91 ± 0.02**^b^** (5) | 1.8E-04 |
| **Lipometabolism indicators** |  |  |  |  |
| total cholesterol (mmol/L) | 5.45 ± 0.16 (41) | 5.53 ± 0.18 (37) | 5.71 ± 0.56 (5) | 9.5E-01 |
| triglyceride (mmol/L) | 1.41 ± 0.11**^a^** (41) | 1.95 ± 0.17**^b^** (37) | 2.63 ± 1.09**^ab^** (5) | 3.2E-02 |
| HDL (mmol/L) | 1.73 ± 0.06**^a^** (41) | 1.42 ± 0.05**^b^** (37) | 1.46 ± 0.07**^ab^** (5) | 9.1E-04 |
| LDL (mmol/L) | 3.14 ± 0.13 (41) | 3.42 ± 0.16 (37) | 3.44 ± 0.4 (5) | 4.0E-01 |
